# Temporally and sex‐specific effects of maternal perinatal stress on offspring cortical gyrification and mood in young adulthood

**DOI:** 10.1002/hbm.25163

**Published:** 2020-10-03

**Authors:** Klara Mareckova, Amy Miles, Lenka Andryskova, Milan Brazdil, Yuliya S. Nikolova

**Affiliations:** ^1^ Campbell Family Mental Health Research Institute Centre for Addiction and Mental Health Toronto Canada; ^2^ Brain and Mind Research Central European Institute of Technology, Masaryk University Brno Czech Republic; ^3^ RECETOX, Faculty of Science Masaryk University Brno Czech Republic; ^4^ Department of Psychiatry University of Toronto Toronto Ontario Canada

**Keywords:** dysregulated mood, gyrification, magnetic resonance imaging, perinatal stress, prenatal birth cohort, sex, young adulthood

## Abstract

Maternal stress during pregnancy and shortly thereafter is associated with altered offspring brain development that may increase risk of mood and anxiety disorders. Cortical gyrification is established during the prenatal period and the first 2 years of life and is altered in psychiatric disorders. Here, we sought to characterize the effects of perinatal stress exposure on offspring gyrification patterns and mood dysregulation in young adulthood. Participants included 85 young adults (56.5% women; 23–24 years) from the European Longitudinal Study of Pregnancy and Childhood (ELSPAC) with perinatal stress data across four distinct timepoints and structural MRI data from young adulthood. Perinatal stress exposure was measured as maternal stress during first and second half of pregnancy, first 6 months, and 6–18 months after birth. Cortical gyrification and mood dysregulation were quantified using local gyrification index (LGI), computed with Freesurfer, and the Profile of Mood States questionnaire, respectively. Perinatal stress predicted cortical gyrification in young adulthood, and its timing influenced location, direction, and sex‐specificity of effects. In particular, whereas early prenatal stress was associated with sex‐dependent medium‐to‐large effects in large temporal, parietal, and occipital regions (*f* ^2^ = 0.19–0.38, *p* < .001), later perinatal stress was associated with sex‐independent small‐to‐medium effects in smaller, more anterior regions (*f* ^2^ = 0.10–0.19, *p* < .003). Moreover, in females, early prenatal stress predicted higher LGI in a large temporal region, which was further associated with mood disturbance in adulthood (*r* = 0.399, *p* = .006). These findings point out the long‐term implications of perinatal stress exposure for cortical morphology and mood dysregulation.

## Graphical abstract

Maternal stress during pregnancy and shortly thereafter is associated with altered offspring brain development that may increase risk of mood and anxiety disorders. Cortical gyrification is established during the prenatal period and the first two years of life and is altered in psychiatric disorders. Here, we sought to characterize the effects of perinatal stress exposure on offspring gyrification patterns and mood dysregulation in young adulthood and demonstrate the long‐term implications of prenatal stress.

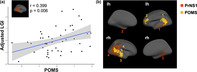

## 1 INTRODUCTION

Exposure to prenatal stress is a global public health problem (Janssen et al., 2016; Kertes et al., 2016; Kinney, Munir, Crowley, & Miller, 2008; Rubin, 2016) reported in 10–35% of children worldwide (Maselko et al., 2015). Prenatal stress alters the structural and functional development of the brain (Mareckova et al., 2019; Mareckova, Klasnja, Andryskova, Brazdil, & Paus, 2019; Sandman, Davis, Buss, & Glynn, 2012; Scheinost et al., 2017), and has been identified as a risk factor for mood and anxiety disorders in adulthood (Brown, van Os, Driessens, Hoek, & Susser, 2000; Mareckova, Klasnja, Andryskova, et al., 2019; Mareckova, Klasnja, Bencurova, et al., 2019; Pearson et al., 2013; van Os, Jones, Lewis, Wadsworth, & Murray, 1997; Watson, Mednick, Huttunen, & Wang, 1999). In addition to these prenatal effects, stress and anxiety experienced by the mother during the early postnatal period have also been tied to more emotional problems in the offspring (Garthus‐Niegel, Ayers, Martini, von Soest, & Eberhard‐Gran, 2017; O'Connor, Heron, Golding, & Glover, 2003; Prenoveau et al., 2017). However, the precise biological mechanisms that mediate these effects remain poorly understood.

Timing of early life stress exposure is critical (Teicher, Samson, Polcari, & McGreenery, 2006; Teicher, Tomoda, & Andersen, 2006), with stress experienced during the first half of pregnancy having particularly strong and long‐lasting impact (Yong Ping et al., 2015). Consistent with these timing effects, maternal anxiety during mid‐pregnancy, but not later, has been associated with decreased gray matter volume in the prefrontal cortex in 6–9‐year‐old children (Buss, Davis, Muftuler, Head, & Sandman, 2010), and research from our group suggests that similar prenatal stress‐related morphological changes may persist into young adulthood (Mareckova, Klasnja, Andryskova, et al., 2019; Mareckova, Klasnja, Bencurova, et al., 2019). Specifically, we recently demonstrated that early prenatal stress, measured prospectively as the number and impact of stressful life events experienced by the mother during the first half of pregnancy, was associated with lower total gray matter (GM) volume as well as lower GM volume in regions implicated in depression (Mareckova, Klasnja, Bencurova, et al., 2019). Further analyses revealed that in one of these regions, namely the mid‐dorsolateral prefrontal cortex, these associations were driven by variations in cortical surface area, rather than cortical thickness (Mareckova, Klasnja, Bencurova, et al., 2019). This finding is consistent with prior longitudinal studies demonstrating substantial increases in cortical surface area but only moderate increases in cortical thickness during the first 2 years of life (Gilmore et al., 2010; Li et al., 2013). It further suggests cortical properties that develop early and remain relatively stable over development may represent a particularly promising substrate linking early life stress to long‐term health outcomes in adulthood.

An important factor that links cortical surface area to cortical complexity and connectivity during development is gyrification. Cortical gyrification of the brain represents the folding of the cerebral cortex, which increases the cortical surface area, and thus the number of neurons in a limited cranium volume (Rakic, 2009). Cortical gyrification is nearly complete by the age of two, and it is mostly unaffected by synaptic pruning and other aging processes (Armstrong, Schleicher, Omran, Curtis, & Zilles, 1995). The degree and complexity of cortical folding can be quantified using the local gyrification index (LGI), a ratio that represents the amount of cortical surface area buried within sulcal folds relative to the amount of cortical surface area visible on the outer surface in a given region (Zilles, Armstrong, Schleicher, & Kretschmann, 1988). Although some studies report age‐dependent decreases in LGI (Cao et al., 2017; Schaer et al., 2008), others suggest that LGI in most brain regions remains relatively stable between the ages of 6 and 30 (Hogstrom, Westlye, Walhovd, & Fjell, 2013). Thus, cortical folding, as measured by LGI, can serve as an index of early brain development, and it can convey information about pre‐ and perinatal developmental disruption, effects of which may persist well into adulthood (Haukvik et al., 2012; Schaer et al., 2008).

Altered LGI has been reported in children (Kesler et al., 2006; Zhang et al., 2015), adolescents (Ganella et al., 2015) as well as adults (Papini et al., 2020) who were born very preterm and the functional consequences of altered LGI has been demonstrated by Papini et al. (2020) who reported association between higher LGI and higher IQ and lower psychopathology scores in both very preterm and control adults. LGI is also altered in psychiatric disorders with a prominent neurodevelopmental component. Specifically, LGI is reduced across multiple regions in patients with schizophrenia (SCZ) and bipolar disorder (BD) (Cao et al., 2017). Notably, however, findings in major depressive disorder (MDD) are more mixed. While some studies did not find any differences in gyrification between MDD and healthy controls (HC) (Cao et al., 2017), others reported both higher (Han et al., 2017; Peng et al., 2015; Schmitgen et al., 2019), and lower (Depping et al., 2018; Nixon et al., 2014; Zhang et al., 2009) gyrification across different brain regions in MDD as compared to HC. Depping et al (Depping et al., 2018) also demonstrated common and distinct patterns of LGI alterations across MDD and BD, suggesting that, rather than being disease‐specific, some LGI abnormalities may mediate broader developmental vulnerability to disorders of emotion (Depping et al., 2018).

Literature also reports sex differences in gyrification (Fish et al., 2017; Li et al., 2014; Raznahan et al., 2011). A longitudinal study of infants from birth to 2 years of age demonstrated that even after the correction for total brain volume, males had significantly larger overall gyrification index than females at the age of 2 and also larger LGI than females in one region at birth and another region at the age of 2 (Li et al., 2014). These findings suggest that sex differences in gyrification exist at birth and continue to develop during the first 2 years of life. Since an independent line of research also reported sex differences in the susceptibility to prenatal influences (Acosta et al., 2019; DiPietro & Voegtline, 2017; Graham et al., 2019; Wen et al., 2017), findings from animal research reported an interference of prenatal stress with typical sexual differentiation (Barrett, Redmon, Wang, Sparks, & Swan, 2014; Bowman et al., 2004; Weinstock, 2007), and further research suggested that mother's exposure to excess glucocorticoids during pregnancy is associated with sex‐dependent impact on the offspring's brain circuitry that regulates mood (Goldstein, Hale, Foster, Tobet, & Handa, 2019), it is likely that the exposure to stress during early life might have different effects on gyrification in men and women.

Taken together, these prior studies suggest that changes in LGI may represent an index of early brain development that could link perinatal stress to psychiatric disorder susceptibility in adulthood. To test this proposition, the current study uses data from a prenatal birth cohort to conduct a prospective evaluation of relationships between exposure to maternal psychosocial stress in utero and early life, as indexed by the number and impact of stressful life events, and cortical gyrification in young adulthood. For the purpose of this study, we quantified maternal stress during four distinct perinatal periods ‐ during the first half of pregnancy, during the second half of pregnancy, during the first 6 months postpartum, and during the subsequent 12 months postpartum. Based on the literature reviewed above, we hypothesized that early life stress exposure would have period‐specific effects on gyrification, with strongest effects occurring during the early prenatal period, independently of stress experienced during the later periods. We also hypothesized that these effects of early life stress exposure on gyrification might differ by sex. Finally, we sought to identify areas wherein gyrification patterns associated with early life stress were also associated with adult mood disturbance.

## 2 MATERIALS AND METHODS

### 2.1 Participants

Participants included young adults (23–24 years old, white Caucasians) from the European Longitudinal Study of Pregnancy and Childhood, Czech Republic (ELSPAC‐CZ; [Piler et al., 2017]), a prenatal birth cohort born in the South Moravian Region of the Czech Republic between 1991 and 1992, who also participated in its neuroimaging follow‐up, “Biomarkers and underlying mechanisms of vulnerability to depression” (VULDE), at the Central European Institute of Technology, Masaryk University. The recruitment flow diagram is provided in Figure S1. While the ELSPAC birth cohort started with the assessments of 5,151 mothers whose children were born between 1991 and 1992, there were only 984 participants with up‐to‐date‐email address from the age of 19 whom we could offer the participation in the neuroimaging follow‐up, a 2‐year Marie Curie individual fellowship (VULDE) aiming to recruit 120 of the ELSPAC participants. We were able to fulfill this goal with 131 MRI assessments. However, only 85 of these individuals with MRI assessment at the age of 23/24 also had complete information from their mothers regarding the exposure to perinatal stress at all four timepoints of interest (see Section 2.2). Therefore, the final sample used for the current gyrification project included 85 individuals (37 male, 48 female) with complete perinatal stress data and adult neuroimaging data were included in the current study. Further characteristics of the sample are provided in Table S1. Approval for the VULDE study was obtained from the ELSPAC Ethics Committee, and all participants provided written informed consent and agreed to merge their VULDE and ELSPAC‐CZ data.

### 2.2 Procedures

Maternal stress was assessed at four time points in the early 1990s: at gestational week 20, shortly after birth, 6 months after birth, and 18 months after birth to assess maternal stress experienced during the first and second half of pregnancy, the first 6 months after birth and during 6–18 months after birth. Maternal stress was indexed using a 40‐item questionnaire (see Supplementary Methods) on which respondents rate their experience of stressful life events (e.g., break up or divorce from partner, consideration of abortion, violence, serious illness or death in the family, financial difficulties) on a 5‐point Likert scale evaluating the presence and impact of the stressful life event on their life (5—it happened and influenced me a lot)/4—happened and influenced me quite a bit/3—happened but influenced me a little/2—happened but did not influence me/1—did not happen) over the period since the beginning of the pregnancy or the most recent assessment. Maternal stress was indexed using a 40‐item questionnaire (see Supplementary Methods) on which respondents ranked their experience of stressful life events (e.g., break up or divorce from partner, consideration of abortion, violence, serious illness or death in the family, financial difficulties) on a 5‐point Likert scale (happened and influenced me a lot/ happened and influenced me quite a bit/ happened but influenced me a little/ happened but did not influence me/ did not happen) over the period since the beginning of the pregnancy or the most recent assessment. Perinatal stress at each time point was quantified as the mean score on this questionnaire. The four time periods sampled were defined as early and late prenatal (PrNS1, PrNS2, corresponding to the first and second half of pregnancy, respectively) and early and late postnatal (PoNS1, PoNS2, corresponding to 0–6 and 6–18 months postpartum, respectively). The means, medians and ranges of the raw early life stress scores by sex are provided in Table S2.

In 2015, structural MRI data was acquired, and participants completed the long version of the Profile of Mood States questionnaire (POMS [McNair, Lorr, & Droppleman, 1971]). This questionnaire measures the following components of current mood state: depression/dejection, tension/anxiety, fatigue/inertia, anger/hostility, confusion/bewilderment, and vigor/activity. As defined in the Manual for the Profile of Mood States (McNair et al., 1971), the total mood dysregulation score was calculated as the sum of the negative mood scores (depression/dejection, tension/anxiety, fatigue/inertia, anger/hostility, confusion/bewilderment) minus the positive mood score (vigor/activity). According to Nyenhuis, Yamamoto, Luchetta, Terrien, & Parmentier (1999), this index of total mood dysregulation shows high and significant correlations with Beck Depression Inventory (BDI; *r* = 0.72), Spielberger's Anxiety Inventory (STAI‐T; *r* = 0.78), as well as the Geriatric Depression Scale (GDS; *r* = 0.82).

### 2.3 MRI acquisition

T1‐weighted images were acquired on 3T Siemens Prisma MRI scanner using a 64‐channel head/neck coil and the following acquisition parameters: voxel size = 1 mm^3^; 240 slices per slab; repetition time (TR) = 2,300 ms; echo time; (TE) > 2.34 ms; inversion time (TI) = 900 ms; flip angle = 8°.

### 2.4 Image processing

The Freesurfer surface‐based processing stream (http://surfer.nmr.mgh.harvard.edu, version 6.0.0), described in detail elsewhere (Dale, Fischl, & Sereno, 1999; Schaer et al., 2008), was used to estimate LGI at each vertex on the cortical surface. In brief, this process involves reconstructing the cortical surface, performing spherical transformation and areal interpolation, and computing LGI as the ratio of surface area buried within sulcal folds to surface area visible on the outer surface. Once computed, LGI values were registered to the Freesurfer average template for subsequent regression analyses, performed using Freesurfer command‐line tools such as mri_glmfit. To ensure accuracy, all reconstructed surfaces were assessed with Freesurfer quality assurance tools, and they were visually inspected by a trained examiner, A.M.

### 2.5 Statistical analysis

In order to test the hypothesis that early life stress exposure would have period‐specific effects on gyrification, with strongest effects occurring during the early prenatal period, independently of stress experienced during the later periods, main effects of period‐specific perinatal stress exposure (PrNS1, PrNS2, PoNS1, PoNS2) on vertex‐wise, cortex‐wide LGI were tested with a single multiple linear regression model simultaneously including all four variables and sex as an additional covariate. The main effect of each period‐specific stress variable *above and beyond* the effects of all remaining period‐specific variables was visualized independently, and correction was applied to account for testing of multiple main effects within the same model (see below). Period‐specific stress‐by‐sex interactions were tested with four additional linear regressions, one per period‐specific variable, using the remaining stress variables as nuisance covariates. Two layers of correction for multiple comparisons, both highly rigorous, were applied to the results. First, to account for repeated testing across the cortex within each analysis, clusters were identified using a Monte Carlo simulation with 10,000 repetitions and vertex‐wise and cluster‐forming thresholds, *p*(*t*)_FWER_ < .05. Second, to account for repeated testing of main and interaction effects across all periods, clusters were retained based on a Bonferroni‐corrected threshold, *p*(*t*)_FWER_ = .05/8 = .00625.

Associations between LGI and POMS total mood disturbance were tested with Pearson correlation. Analyses were repeated for each cluster in which we detected a significant main effect of stress exposure on LGI. Significance of associated correlation coefficients was determined using false discovery rate (*p*_FDR_ < .05). This method of multiple comparison correction, less conservative than the Bonferroni method previously employed, was selected due to the follow‐up nature of this analysis and to its relative simplicity compared to the aforementioned vertex‐wise analysis, which featured repeated testing in spatial and temporal domains (i.e., across the cortex and across perinatal periods) and for which more stringent correction methods were deemed necessary. Given the anticipated high correlation among stress variables and associated concerns about regression multicollinearity, we computed tolerance and variance inflation factor (VIF) values for each stress‐associated cluster. Consistent with prior published work, tolerance values >0.1 and VIF < 10 (Tabachnick & Fidell, n.d.) were considered acceptable.

All statistical analyses were performed using RStudio (RStudio Team, version 0.99.489). Given significant positive skewness (*b*1 = 0.7–2.1), a Yeo‐Johnson transformation was applied to stress and mood variables in order to improve normality (*b*1 = 0.0–0.3). The Yeo‐Johnson transformation, an extension of the Box‐Cox transformation, is a power transformation, the aim of which is to stabilize variance and improve normality (Weinberg, 2001).

## 3 RESULTS

### 3.1 Temporally specific associations between perinatal stress exposure and LGI

**FIGURE 1:**
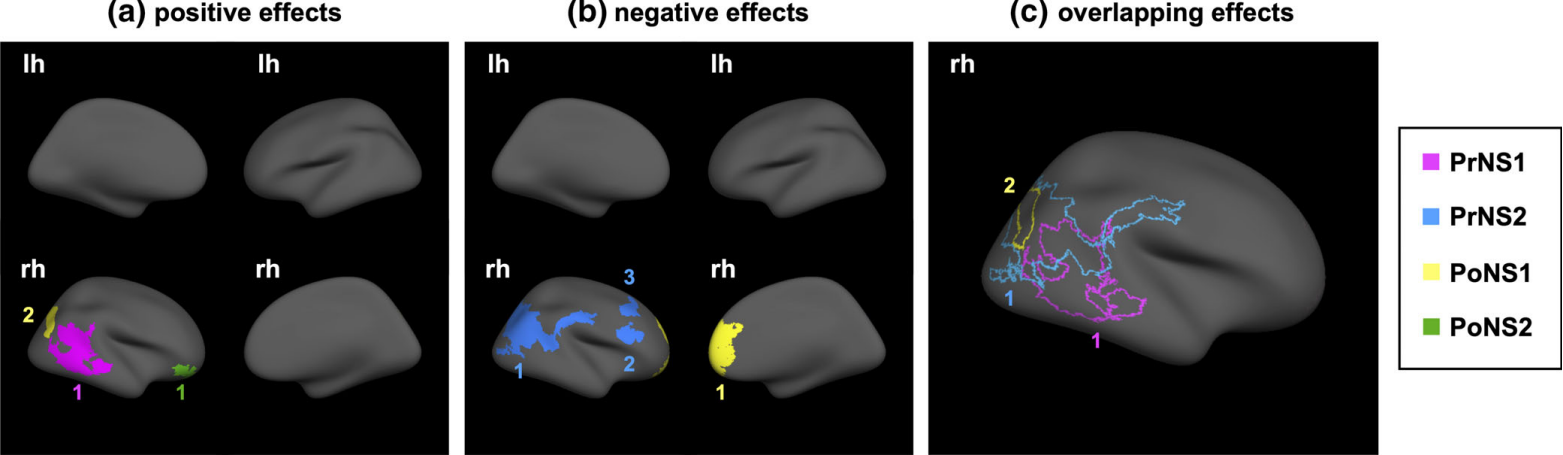
Sex‐independent effects of early life stress on vertex‐wise LGI. Positive (a), negative (b), and overlapping (c) main effects of period‐specific perinatal stress exposure on vertex‐wise LGI. (PrNS1, stress experienced by the mother during first half of pregnancy; PrNS2, stress experienced by the mother during second half of pregnancy; PoNS1, stress experienced by the mother during first 6 months postpartum; PoNS2, stress experienced by the mother during months 6–18 postpartum; lh, left hemisphere; rh, right hemisphere)

We detected significant effects of perinatal stress exposure on LGI in 12 distinct and overlapping clusters spanning parts of the frontal, temporal, and parietal cortices (Figure 1 and Table S3). Despite the moderate to high correlation among the four distinct perinatal variables (*r* = 0.45–0.68, *p* < .001; Figure S2), their effects were clearly dissociable and varied in size, direction and sex‐dependence. Tolerance and VIF values for all identified clusters were within the acceptable range, suggesting no multicollinearity concerns (Table S3).

Across analyses, earlier stress was generally associated with larger cluster sizes (Table S3). Combined, period‐specific clusters covered 20% (PrNS1), 10% (PrNS2), 5% (PoNS1), and 1% (PoNS2) of vertices on the cortical surface. Likewise, cluster location varied as a function of perinatal period (Figure 1); exposure to maternal stress during the early prenatal period was primarily associated with LGI in temporal, parietal, and occipital regions, while stress exposure during the late prenatal and early postnatal periods was associated with LGI in parietal and frontal regions. Finally, exposure to maternal stress during the late postnatal period (at 18 months of age) was associated with LGI in a region overlapping the pars orbitalis and lateral orbitofrontal cortex.

Direction of effect also varied as a function of perinatal period (Figure 1). Unlike cluster size and location, however, this effect was nonlinear, shifting between positive (PrNS1, PoNS2) and negative (PrNS2, PoNS1). Thus, across distinct regions, maternal stress experienced more distal to birth (i.e., during the first half of pregnancy and at 18 months of age) was generally associated with higher gyrification, while maternal stress experienced closer to birth (i.e., in the second half of pregnancy and at 6 months postpartum) was associated with lower gyrification.

### 3.2 Sex‐specific associations between early prenatal stress and LGI

**FIGURE 2:**
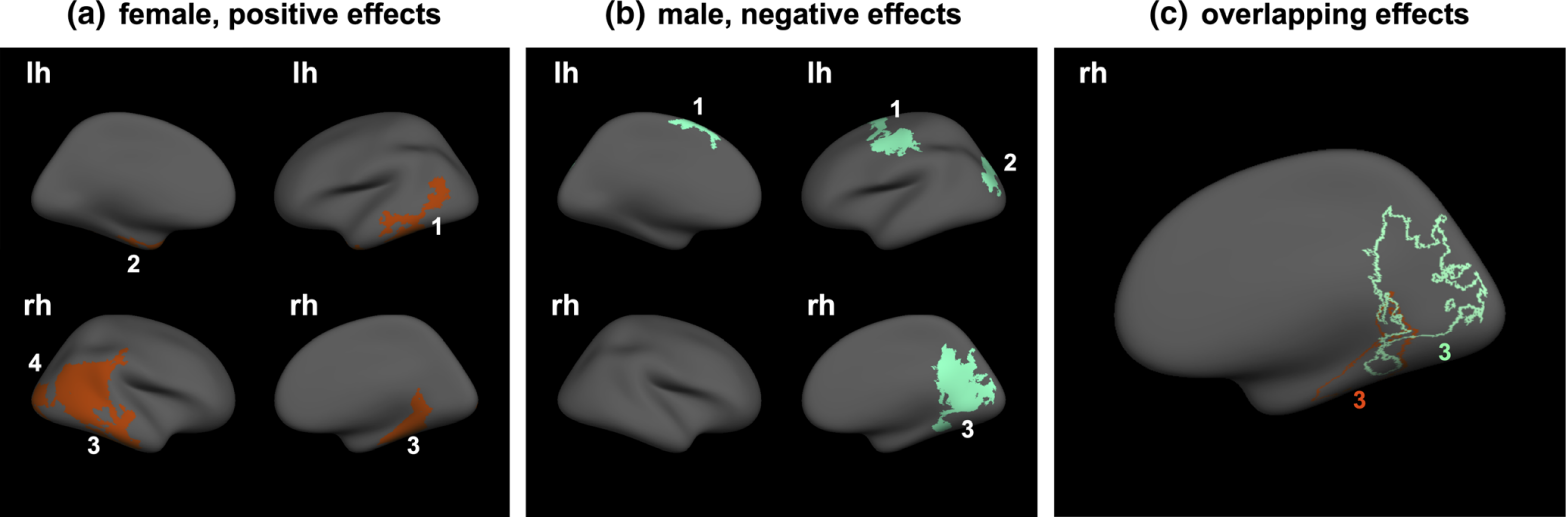
Sex‐specific effects of stress experienced by the mother during first half of pregnancy on vertex‐wise LGI. Female‐specific (a), male‐specific (b), and overlapping (c) main effects of early prenatal stress exposure on vertex‐wise LGI. (positive effects—higher stress associated with higher LGI; negative effects—higher stress associated with lower LGI)

Remarkably, the early prenatal period was the only one in which effects of stress exposure showed sex‐specificity in terms of both direction and location of effect. During this period, we detected significant interaction effects of sex and stress exposure on LGI in five clusters spanning parts of the frontal, temporal, and occipital cortices (Figure S3 and Table S3). In order to shed light on any sex‐dependent effects, we ran additional vertex‐wise analyses testing the effects of early prenatal stress exposure on LGI in female and male participants separately (Figure 2 and Table S4). In females, early prenatal stress was associated with greater gyrification in occipito‐temporal regions (Figure 2a and Table S4). In males, by contrast, early prenatal stress was associated with reduced gyrification in nonoverlapping frontal and parieto‐occipital regions and in an overlapping medial occipital region (Figure 2b,c and Table S4). In the latter region, prenatal stress exerted opposite effects on LGI in males and females.

### 3.3 LGI and mood disturbance

**FIGURE 3:**
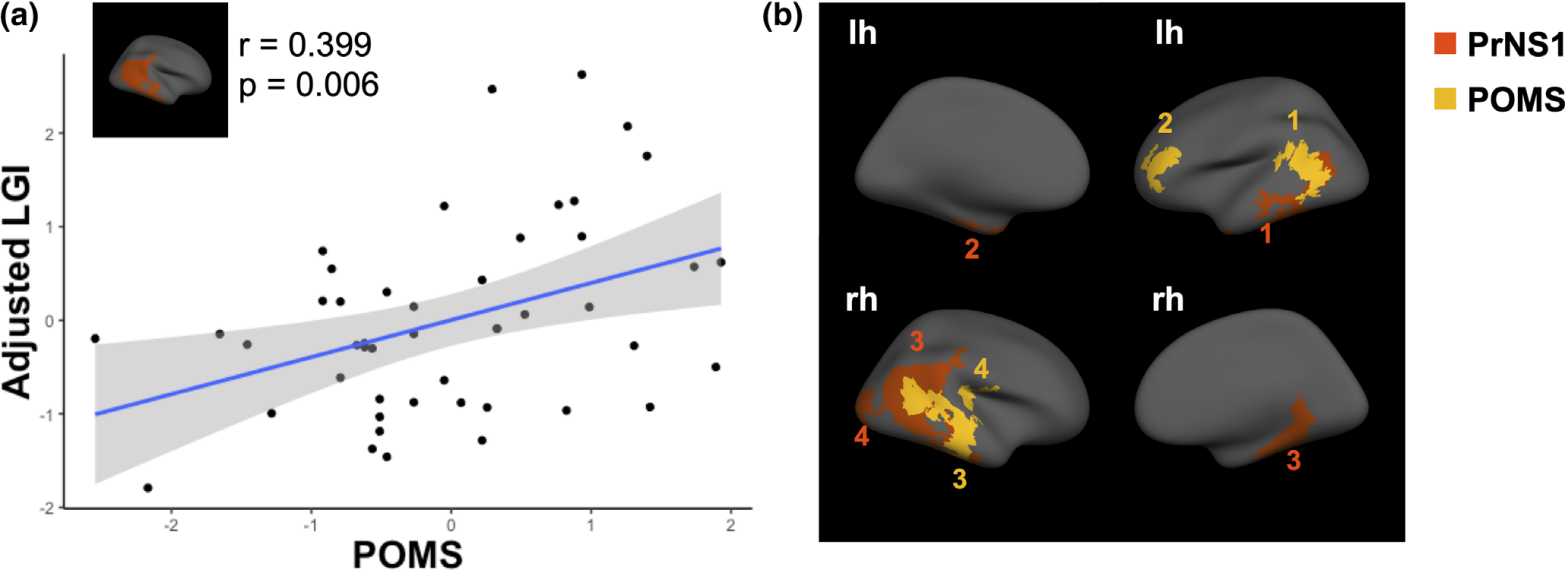
Overlapping effects of early prenatal stress and total mood disturbance. Correlation between total mood disturbance (POMS) and LGI in an early prenatal stress‐associated cluster in females (a). Clusters in which total mood disturbance was a significant predictor of vertex‐wise LGI in females, overlaid onto clusters in which early prenatal stress exposure is a significant predictor of vertex‐wise LGI in females (b)

We sought to further map these stress‐associated LGI patterns onto mood disturbance in young adulthood by correlating total POMS scores with extracted LGI values from all 14 clusters (7 sex‐independent and 7 sex‐dependent) in which we identified a significant main effect of period‐specific stress on LGI. We detected a positive association between total mood disturbance and LGI in a right temporal region (*r* = 0.399, *p* = .006), wherein higher LGI was also associated with early prenatal stress exposure in females (Figure 3a). However, this effect was reduced to a trend when correction for multiple comparisons across all 14 clusters was applied (*p*_FDR_ = .084). Hence, we conducted additional vertex‐wise analyses evaluating the link between POMS and LGI in females in order to assess overlap in emerging clusters independently associated with stress and mood disturbance. We observed significant overlap in a right temporal region, where higher LGI may represent a common substrate linking early prenatal stress with mood symptoms in adult females. POMS was additionally associated with higher LGI in clusters spanning left frontal, left temporal, and right parieto‐temporal regions (Figure 3b and Table S5).

## 4 DISCUSSION

We conducted a neuroimaging follow‐up of a prenatal birth cohort to study the impact of maternal stress, measured repeatedly during pregnancy and the first 18 months after birth, on gyrification of the brain in young adult offspring. We found that gyrification of the brain in young adulthood was associated with maternal stress and that the timing of the stress exposure influenced location, directionality and sex‐specificity of these effects, as well as their relevance to symptoms of mood dysregulation in young adulthood (age 23–24). The focus on the impact of stressful life events experienced by the mother during four distinct perinatal periods spanning the whole pregnancy and first 18 months of the offspring's life on gyrification and the use of vertex‐wise approach substantially extend our initial findings from the same cohort on the impact of stressful life events during first half of pregnancy on GM volume (Mareckova, Klasnja, Bencurova, et al., 2019) and are particularly striking given that the maternal stress measures reflected the experience of relatively common stressful life events.

The fact that prenatal stress exposure affected gyrification more extensively than postnatal maternal stress is consistent with normal trajectories of cortical folding. Previous research by Nishikuni and Ribas (2013) demonstrated that all primary sulci are formed during the fetal stage. Gyrification starts as early as the first half of pregnancy, namely between 10th and 15th week of gestation (Chi, Dooling, & Gilles, 1977; Zilles et al., 1997), and undergoes the greatest development during the third trimester of fetal life (White, Su, Schmidt, Kao, & Sapiro, 2010), when the brain is undergoing considerable growth (Chi et al., 1977). Postmortem studies (Armstrong et al., 1995; Zilles et al., 1988; Zilles, Palomero‐Gallagher, & Amunts, 2013) as well as longitudinal imaging studies in children before the age of 2 (Li et al., 2014) agree that gyrification continues to increase after birth, albeit at a slower pace.

While prenatal exposure to maternal stress was associated with altered gyrification in temporal regions, postnatal exposure to maternal stress was primarily associated with altered gyrification in frontal regions. Similar time‐ and location‐ specific effects of stress on the developing brain were reported also by animal research which demonstrated effect of early life stress on reduced synaptic density in medial temporal lobe structures but of later stress on reduced synaptic density in the prefrontal cortex (Andersen & Teicher, 2004, 2008). These time and location‐specific effects of stress on gyrification are also broadly consistent with the time and location‐specific trajectories of gyrification development (White et al., 2010).

Interestingly, the effects of maternal stress experienced during the first half of pregnancy (but not later) on gyrification also differed by the sex of the offspring. While higher stress exposure during the first half of pregnancy was associated with less gyrification in young adult men, particularly in the superior frontal, inferior parietal and pericalcarine clusters, higher stress exposure during the first half of pregnancy was associated with more gyrification in young adult women, particularly in the temporal pole and across inferior temporal, middle temporal and lateral occipital clusters. It might be that this opposite directionality of the effects of stress during first half of pregnancy in men vs. women might be driven by the interference of prenatal stress with typical sexual differentiation reported in animal literature (Barrett et al., 2014; Bowman et al., 2004; Weinstock, 2007). While our previous research in this prenatal birth cohort did not find any sex differences in the effect of prenatal stress experienced during the first half of pregnancy on gray (Mareckova, Klasnja, Bencurova, et al., 2019) or white (Mareckova, Klasnja, Andryskova, et al., 2019) matter, the current findings from the large subsample of these studies suggest that the different hormonal milieu of the male vs. female fetus might moderate the effects of maternal stress on gyrification, independently of other structural brain properties. In fact, sex‐specific effects of prenatal stress on the brain have been reported by a number of studies (Bock, Wainstock, Braun, & Segal, 2015; Buss, Entringer, Swanson, & Wadhwa, 2012; Gilman et al., 2016). The majority of studies in both animals (Behan et al., 2011; Schroeder, Sultany, & Weller, 2013) and humans (Buss et al., 2012) report effects of prenatal stress on anxiety and depressive‐like behavior in females. Future research should determine whether the sex‐specific effects of prenatal stress on gyrification observed in our study might be related to sex‐specific placental adaptation to stress exposure (Clifton, 2010) or more rapid neurodevelopmental trajectory in females vs. males (Buss et al., 2009; Nathanielsz et al., 2003).

In females, gyrification was also associated with dysregulated mood, as measured with the POMS questionnaire. Females with more dysregulated mood had more gyrification in the right middle temporal cortex, a cluster that was also associated with higher exposure to prenatal stress during the first half of pregnancy. Although the link between LGI in stress‐associated cluster was only associated with POMS at a trend‐level after FDR correction (*p*_FDR_ = .084), a vertex‐wise analysis with POMS, showed substantial overlap in the temporal lobe clusters wherein greater gyrification was independently associated with prenatal stress and mood disturbance. These findings are partly consistent with previous research which found greater gyrification of the temporal cortex, as well as other regions, in patients with generalized anxiety disorder (Molent et al., 2018) and major depressive disorder (Schmitgen et al., 2019). Taken together, these findings suggest that altered gyrification in the temporal lobe may link early life stress to potentially elevated risk of mood disorders in adult females. Interestingly, no relationships between dysregulated mood and gyrification were found in males. These sex differences in behavioral outcomes might be, in part, explained by the fact that the female brain is more susceptible to insults than the male brain (Buss et al., 2009; Nathanielsz et al., 2003) and by the fact that mood disorders are generally more prevalent in women vs. men (Kessler et al., 2005; Kornstein et al., 2000).

Cortical folding comprises a complex sequence of events that include neurogenesis, cell migration, axon and dendrite growth, as well as the generation of synapses. Each of these processes has its own distinct developmental dynamics and is regulated by growth factors with distinct expression patterns across brain regions and developmental stages (Sun & Hevner, 2014). Thus, we speculate that the spatiotemporal specificity of the effects we observed is likely driven by different cellular or growth factor mechanisms, with prenatal effects primarily mediated by alterations in neurogenesis and cell migration, and postnatal effects mediated primarily by alterations in neurite growth and synapse formation. Consistent with this conjecture, macaques exposed to prenatal stress had increased levels of basal cortisol and reduced neurogenesis (Pryce, Aubert, Maier, Pearce, & Fuchs, 2011) and administration of synthetic glucocorticoid dexamethasone to pregnant rats resulted in delayed maturation of neurons and inhibition of neurogenesis in the offspring (Lupien, 2009). Early postnatal stress has been associated with both increased (Helmeke, Ovtscharoff Jr., Poeggel, & Braun, 2001; Monroy, Hernandez‐Torres, & Flores, 2010) and decreased (Bock, Gruss, Becker, & Braun, 2005; Monroy et al., 2010; Pinzon‐Parra et al., 2019) neuronal complexity and synaptic density in a regionally specific manner. Given the lack of cortical folding in rodents, parallel studies in experimental models and human postmortem tissue may be needed to validate the contribution of these putative molecular mechanisms to altered gyrification patterns.

The current study has a number of strengths. First, it was designed as a neuroimaging follow‐up of a prenatal birth cohort, which means that our relatively large sample size consisted of individuals from a very similar background (all White Caucasians, birth weight within the normal range, typically developing, growing‐up in the same area and due to the early postcommunist era in Czechoslovakia in the early 90s, they were born into families with very similar socioeconomic status) and a very narrow age range (23 or 24 years old), thus eliminating any potential age or birth‐related effects on gyrification. Albeit it is also possible to see this strength as a limitation since such specificity of the sample might contribute to possible inconsistencies with existing literature that might contain more mixed samples. Second, the unique data from European Longitudinal Study of Pregnancy and Childhood (ELSPAC‐CZ) allowed us to use prospective measures of maternal stress measured repeatedly during pregnancy and the first 18 months of life, which are known to be critical for gyrification of the brain, and evaluate their time‐specific effects. Third, all measures of prenatal and early postnatal stress were based on data collected in the early 90s, thus eliminating any possible false memories and recall bias. Fourth, we also assessed any possible interactions with sex, which have been reported in the context of previous research on the impact of prenatal stress. Still, given the complexity of the findings, the lack of replication limits our conclusions and should be conducted by future research in another prenatal birth cohort with the same design. Further, since our sample was overall comparable to nonclinical populations (Nyenhuis et al., 1999), future research might also consider a replication of our findings in a clinical sample with more dysregulated mood.

### 4.1 Conclusions

We conclude that the exposure of immature brain to maternal stress has long‐term implications for cortical morphology and dysregulated mood and that the timing of these effects is essential for the location, directionality and sex‐specificity of these effects. These findings may have important implications for understanding depression pathophysiology and may open novel opportunities for developing early individualized intervention and prevention strategies.

## Supporting information


**Data S1** Supplementary Information.

**Figure S1** Recruitment flow diagram.

**Figure S2** Correlations between stress exposure during each perinatal period of interest.

**Figure S3** Sex moderates the impact of early prenatal stress on vertex‐wise LGI. Stress‐by‐sex interaction effects (a) and sex‐specific correlations (b–f) between early prenatal stress exposure and cluster‐wise LGI, adjusted for other perinatal stress exposure.

## Data Availability

The data that support the findings of this study are available from the corresponding author upon reasonable request.
