# Supplementary material for: Temporally and sex‐specific effects of maternal perinatal stress on offspring cortical gyrification and mood in young adulthood
Source: Hum Brain Mapp. 2020 Oct 3;41(17):4866–75. doi: 10.1002/hbm.25163 (PMC7643354; doi:10.1002/hbm.25163)

**SUPPLEMENTARY INFORMATION**

**Supplementary Methods**

**List of the 40 questions included in the Stressful Life Events questionnaire and answered on a 5-point Likert scale regarding the presence and impact of the stressful experience.**

1. Death of your partner.
2. Death of one of your children.
3. Death of your friend or relative.
4. One of your children was ill.
5. One of your partners was ill.
6. One of your friends or relatives was ill.
7. You were hospitalized.
8. You had legal issues.
9. You got divorced.
10. You found out your partner does not want your child.
11. You were seriously ill.
12. Your partner lost a job.
13. Your partner had problems in his job.
14. You had problems in your job.
15. You lost your job.
16. Your partner broke up with you.
17. Your partner had legal issues.
18. You broke up with your partner.
19. Your income decreased.
20. You argued with your partner.
21. You had an argument with your family or friends.
22. You moved.
23. Your partner hurt you physically.
24. You lost your accommodation.
25. You had serious financial difficulties.
26. You got married.
27. Your partner physically hurt your children.
28. You tried to kill yourself.
29. You were convicted of violating a law.
30. You were bleeding and thought you might lose your child.*
31. You started a new job.
32. You were on an examination whether your child might have any defects.*
33. You found out your child might not be completely healthy.
34. You found out that something happened that might hurt the health of your child.*
35. You tried to abort your pregnancy.
36. You took an exam.
37. Your partner was emotionally cruel to you.
38. Your partner was emotionally cruel to your children.
39. Somebody robbed your apartment/house or a car.
40. You had an injury.

- Stressful life events marked with a star where applicable only for the pregnancy period and thus the stressful life events questionnaire administered postnatally replaced these three items with the following stressful life events: You returned to your job. You got pregnant. You experienced a spontaneous abortion.

**Supplementary Results**

**Supplementary Table 1. Demographics Table.**

|  | **Males**  (n=37) | **Females** (n=48) | **Between group difference** |
| --- | --- | --- | --- |
| **Ethnicity**  % White Caucasian | 100% | 100% | No difference. |
| **Age**  in years (M, SD) | M=23.88, SD=0.38 | M=23.77, SD=0.37 | No difference. |
| **Handedness**  % Right-handed | 86.49% | 89.58% | No difference. |
| **BMI** (M, SD) | M=24.12, SD=3.80 | M=22.60, SD=2.91 | Males > Females |
| **Education**  % completed elementary school  % completed high school  % completed undergraduate degree  % completed masters degree | 2.70%  59.46%  29.73%  8.11% | 2.08%  58.33%  39.58%  0% | No difference. |
| **Birthweight**  in grams (M, SD) | M=3482.43, SD=560.41 | M=3302.08, SD=464.48 | Males > Females |
| **Maternal Age at Birth**  in years (M, SD) | M=27.97, SD=5.09 | M=27.25, SD=4.87 | No difference. |
| **Maternal Education**  % not completed high school  % completed high school  % completed university  % completed postgraduate educ.  % missing | 13.51%  32.43%  45.95%  5.41%  2.70% | 20.83%  41.67%  29.16%  4.17%  4.17% | No difference. |
| **Maternal Smoking**  % not smoking during pregnancy  % smoking during pregnancy  % missing | 94.60%  2.70%  2.70% | 97.92%  2.08%  0% | No difference. |

**Supplementary Table 2 – Means, medians and ranges of the raw early life stress and POMS variables by sex.**

| **Variable** | **Males** | | | **Females** | | |
| --- | --- | --- | --- | --- | --- | --- |
|  | **Mean** | **Median** | **Range** | **Mean** | **Median** | **Range** |
| **PrNS1** | 0.24 | 0.21 | 0 - 0.88 | 0.20 | 0.18 | 0 - 0.83 |
| **PrNS2** | 0.17 | 0.15 | 0 - 0.61 | 0.17 | 0.12 | 0 - 0.59 |
| **PoNS1** | 0.19 | 0.17 | 0 - 0.56 | 0.17 | 0.14 | 0 - 0.51 |
| **PoNS2** | 0.27 | 0.21 | 0 - 1.12 | 0.25 | 0.19 | 0 - 1.12 |
| **POMS** | 21.05 | 15 | -17 - 104 | 23.83 | 14 | -18 - 135 |

**Supplementary Table 3. Effects of period-specific perinatal stress on LGI**

| Cluster | Max | CWP | Size (mm^2^) | Annot. | f^2^ |
| --- | --- | --- | --- | --- | --- |
| PrNS1 (VIF = 2.2, tolerance = 0.455) | | | | | |
| 1 | 4.578 | <0.001 | 3844 | middle temporal, R | 0.141 |
| 2 * | -4.164 | <0.001 | 6598 | inferior parietal, L | n/a |
| 3 * | -2.323 | <0.001 | 1079 | superior frontal, L | n/a |
| 4 * | -3.879 | <0.001 | 5904 | isthmus cingulate, R | n/a |
| 5 * | -2.071 | <0.001 | 1048 | precentral, R | n/a |
| 6 * | -2.547 | 0.005 | 727 | lateral occipital, R | n/a |
| PrNS2 (VIF = 1.9, tolerance = 0.526) | | | | | |
| 1 | -5.162 | <0.001 | 6261 | inferior parietal, R | 0.186 |
| 2 | -3.618 | 0.001 | 847 | pars opercularis, R | 0.099 |
| 3 | -3.874 | 0.002 | 819 | caudal middle frontal, R | 0.119 |
| PoNS1 (VIF = 2.5, tolerance = 0.400) | | | | | |
| 1 | -3.166 | <0.001 | 3755 | superior frontal, R | 0.102 |
| 2 | 3.026 | <0.001 | 1169 | superior parietal, R | 0.103 |
| PoNS2 (VIF = 1.6, tolerance = 0.625) | | | | | |
| 1 | 3.295 | 0.003 | 786 | pars orbitalis, R | 0.116 |

Clusters in which we detected significant main or interaction effects of period-specific perinatal stress exposure on LGI.

** stress-by-sex interaction effect; Annot. = Desikan-Killiany atlas-based parcellation in which the peak vertex is located; CWP = cluster-wise p-value; L = left hemisphere; Max = T statistic at peak vertex; R = right hemisphere*

**Supplementary Table 4. Sex-specific effects of early prenatal stress on LGI**

| Cluster | Max | | CWP | | Size (mm^2^) | | Annot. | | f^2^ |
| --- | --- | --- | --- | --- | --- | --- | --- | --- | --- |
| Female (VIF = 2.3, tolerance = 0.435) | | | | | | | | | |
| 1 | | 3.548 | | <0.001 | | 2479 | | inferior temporal, L | 0.261 |
| 2 | | 3.241 | | <0.001 | | 1205 | | temporal pole, L | 0.211 |
| 3 | | 5.327 | | <0.001 | | 9747 | | middle temporal, R | 0.381 |
| 4 | | 2.763 | | <0.001 | | 1399 | | lateral occipital, R | 0.188 |
| Male (VIF = 2.0, tolerance = 0.500) | | | | | | | | | |
| 1 | | -2.866 | | <0.001 | | 3182 | | superior frontal, L | 0.224 |
| 2 | | -3.570 | | <0.001 | | 1423 | | inferior parietal, L | 0.272 |
| 3 | | -2.789 | | <0.001 | | 4356 | | pericalcarine, R | 0.206 |

Clusters in which we detected significant main effects of early prenatal stress exposure on LGI when tested separately in females and males.

*Annot. = Desikan-Killiany atlas-based parcellation in which the peak vertex is located; CWP = cluster-wise p-value; L = left hemisphere; Max = T statistic at peak vertex; R = right hemisphere*

**Supplementary Table 5. Effects of total mood disturbance on LGI**

| Cluster | Max | CWP | Size (mm2) | Annot. |
| --- | --- | --- | --- | --- |
| 1 | 3.799 | <0.001 | 2021 | middle temporal, L |
| 2 | 3.752 | <0.001 | 1369 | rostral middle frontal, L |
| 3 | 3.586 | <0.001 | 3166 | middle temporal, R |
| 4 | 3.082 | 0.005 | 712 | supramarginal, R |

Clusters in which we detected significant main effects of total mood disturbance on LGI when tested in females.

*Annot. = Desikan-Killiany atlas-based parcellation in which the peak vertex is located; CWP = cluster-wise p-value; L = left hemisphere; Max = T statistic at peak vertex; R = right hemisphere*

**Supplementary Figure 1. Recruitment flow diagram**

**

**

**Supplementary Figure 2. Correlations between stress exposure during each perinatal period of interest.**

**
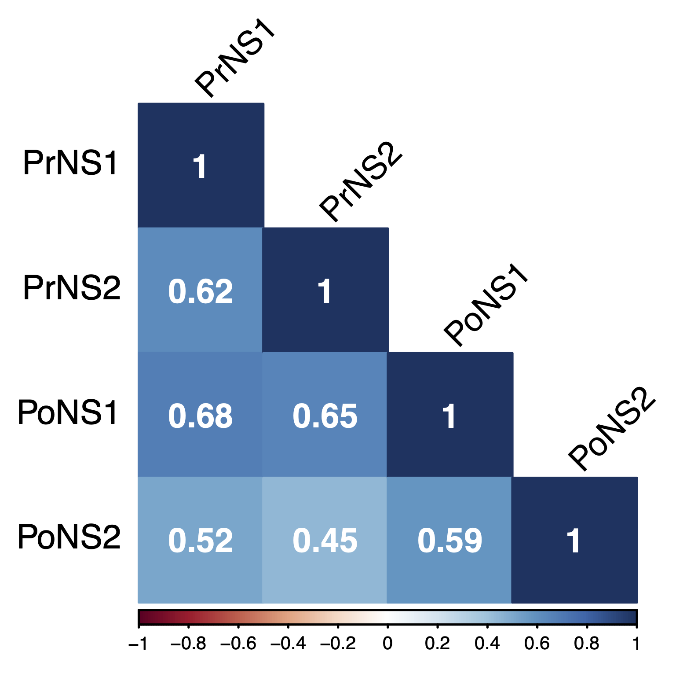
**

**Supplementary Figure 3. Sex moderates the impact of early prenatal stress on vertex-wise LGI.** Stress-by-sex interaction effects **(3A)** and sex-specific correlations **(3B, 3C, 3D, 3E, 3F)** between early prenatal stress exposure and cluster-wise LGI, adjusted for other perinatal stress exposure.


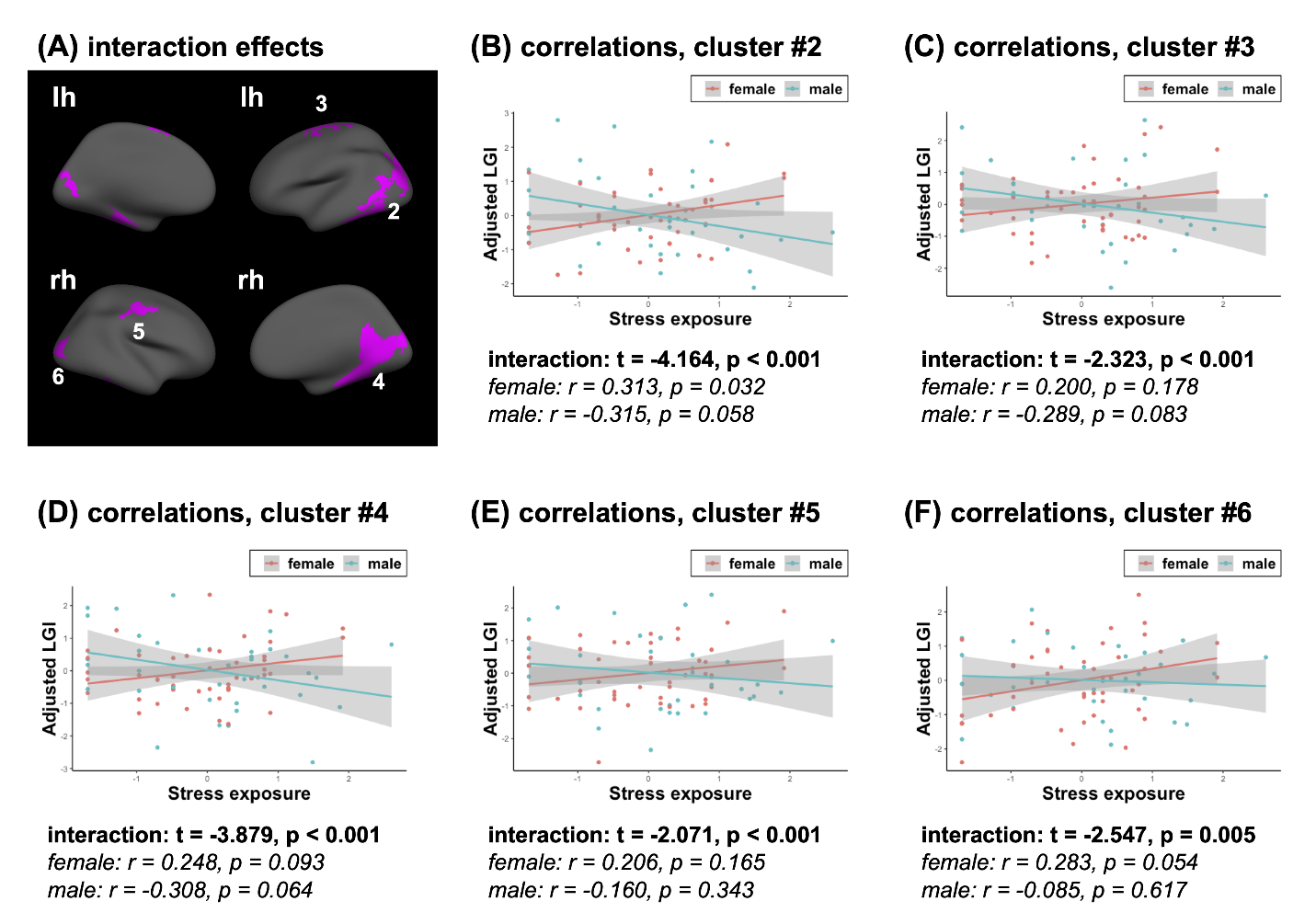

Supplement: Supplementary file 1 — Data S1 Supplementary Information. [file HBM-41-4866-s001.docx]
