# Supplementary figures and images for: Temporally and sex‐specific effects of maternal perinatal stress on offspring cortical gyrification and mood in young adulthood
Source: Hum Brain Mapp. 2020 Oct 3;41(17):4866–75. doi: 10.1002/hbm.25163 (PMC7643354; doi:10.1002/hbm.25163)

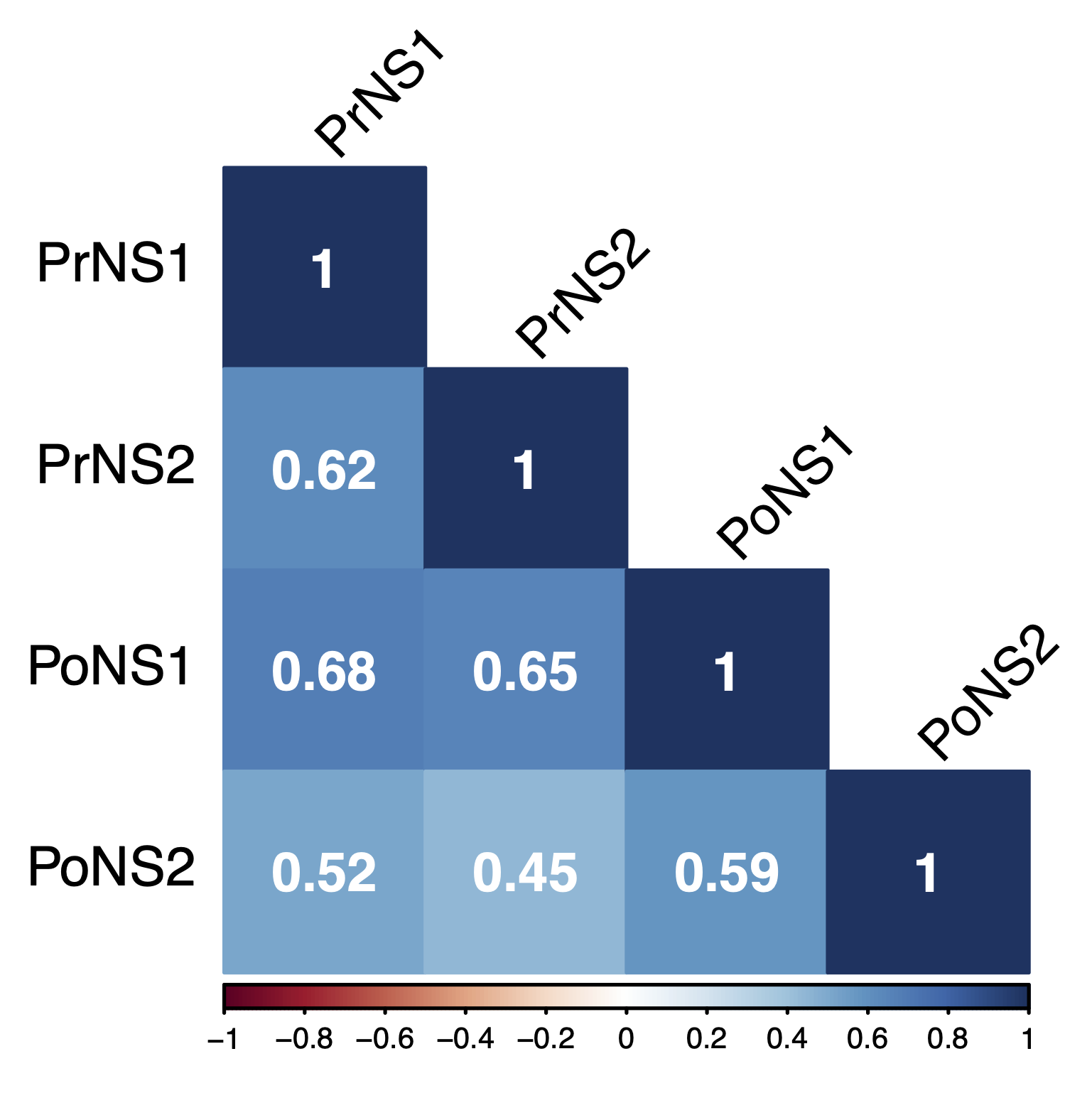

Supplement: Supplementary file 3 — Figure S2 Correlations between stress exposure during each perinatal period of interest. [file HBM-41-4866-s003.tiff]

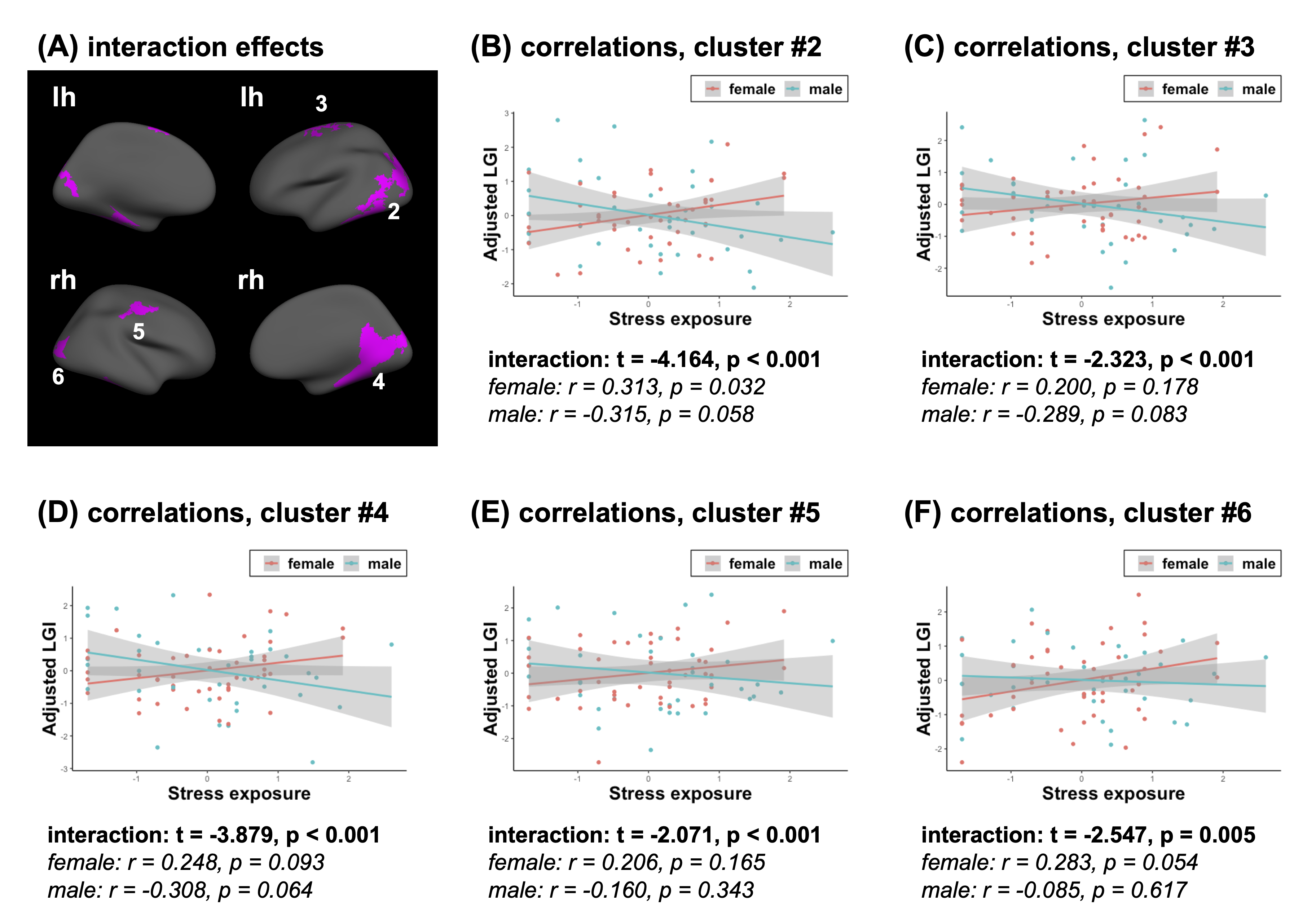

Supplement: Supplementary file 4 — Figure S3 Sex moderates the impact of early prenatal stress on vertex‐wise LGI. Stress‐by‐sex interaction effects (a) and sex‐specific correlations (b–f) between early prenatal stress exposure and cluster‐wise LGI, adjusted for other perinatal stress exposure. [file HBM-41-4866-s004.tiff]
